# Supplementary figures and images for: Limits of life: Thermal tolerance of deep-sea hydrothermal vent copepods and implications for community succession
Source: PLoS One. 2025 Nov 5;20(11):e0333996. doi: 10.1371/journal.pone.0333996 (PMC12588466; doi:10.1371/journal.pone.0333996)

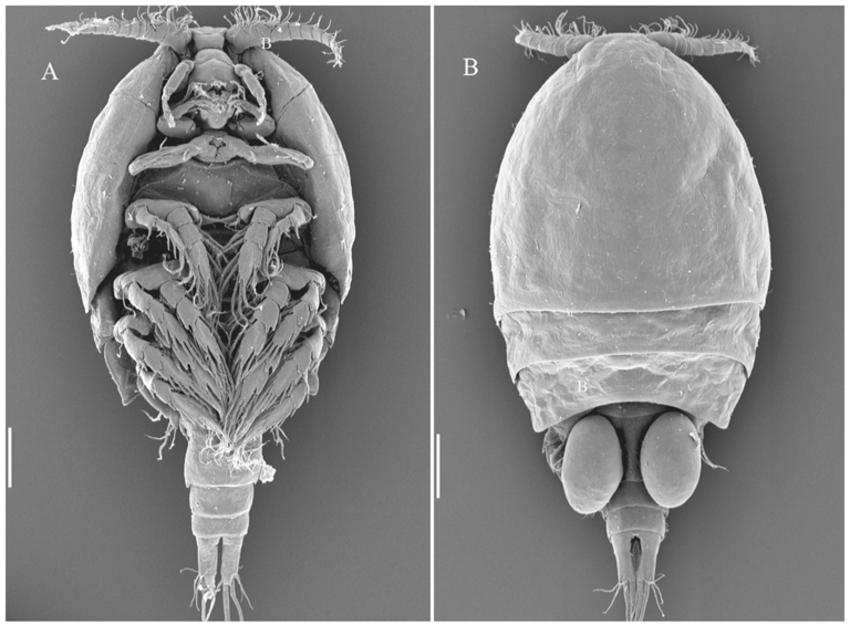

Supplement: S1 Fig — Stygiopontius pectinatus female SEM micrographs. A: ventral view. B: dorsal view. Scale bars 100 µm. Images originally from [38] and adapted in [45]. (TIF) [file pone.0333996.s001.tif]

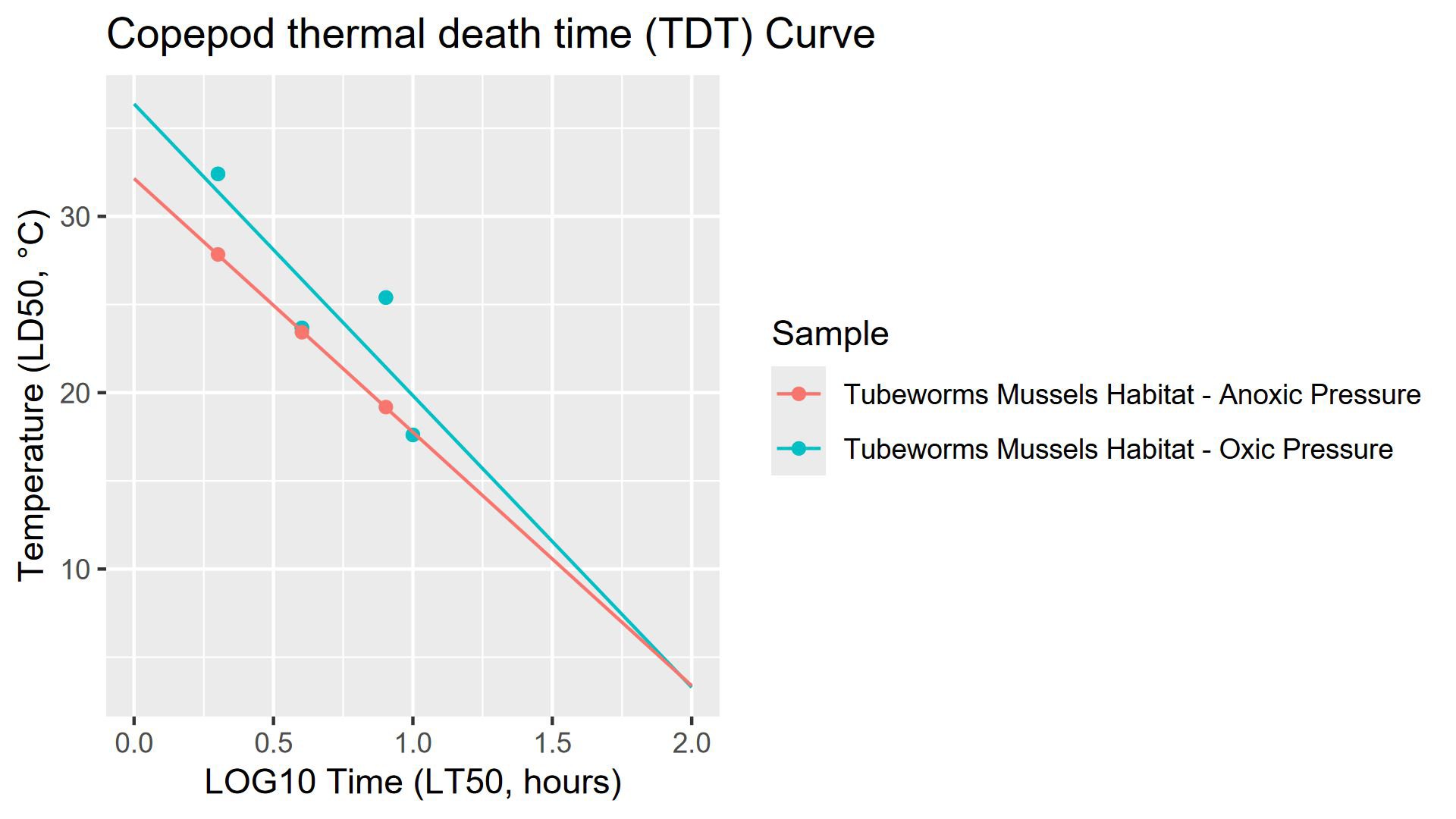

Supplement: S2 Fig — Incubations were performed at 200 bar. Points represent LD50 values extrapolated from the survival curves. Tubeworm & mussel copepods in anoxic conditions (red line) have CTmax of 32.1 ± 0.1°C and slope −14.4 ± 0.2. Tubeworm & mussel copepods in oxic conditions (blue line) have CTmax of 36.4 ± 5.2°C and slope −16.5 ± 6.9. For curve fitting statistics see Table 2. (TIF) [file pone.0333996.s002.tif]

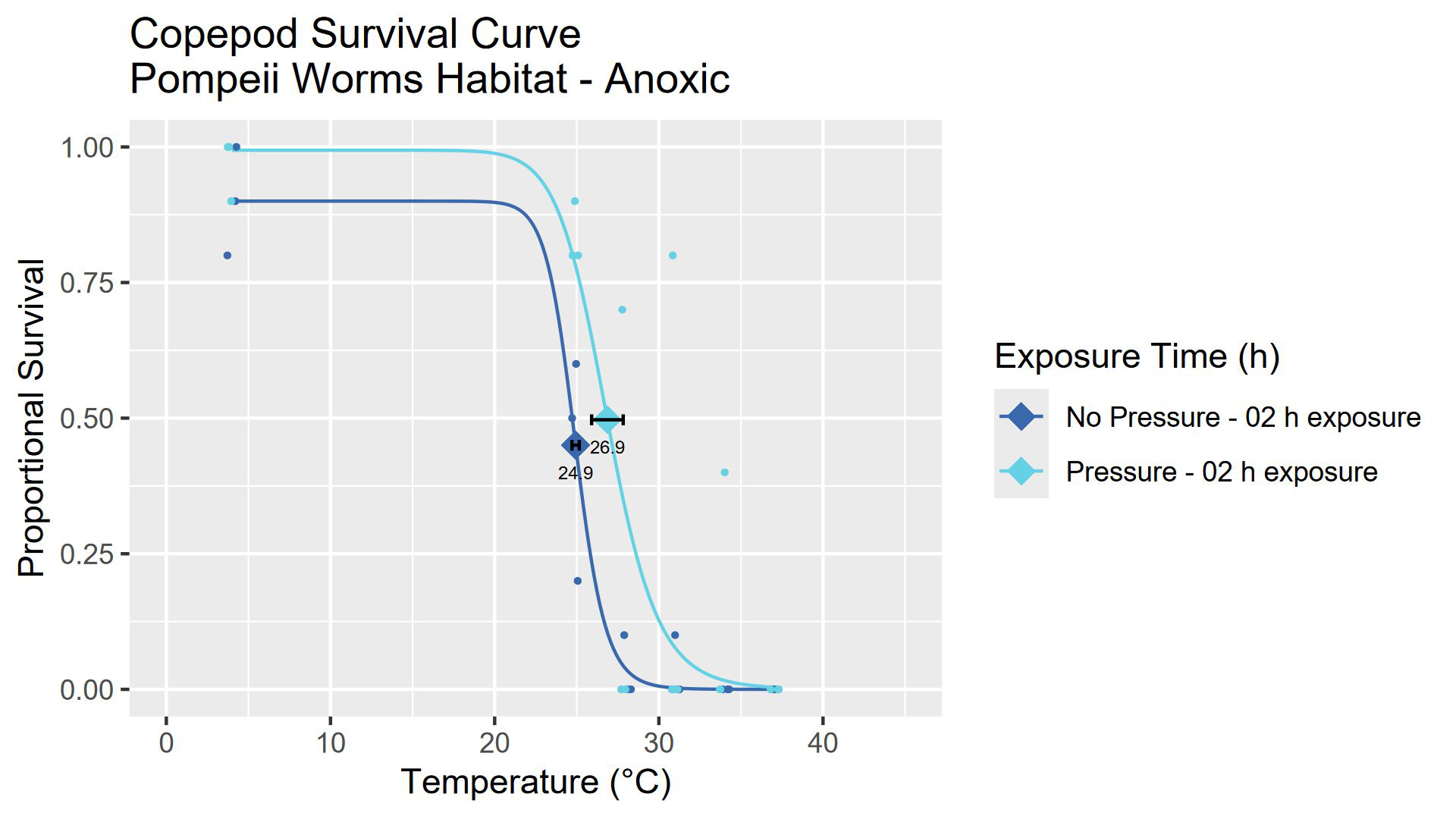

Supplement: S3 Fig — LD50 values with standard error bars are marked with a diamond shape. Proportional survival given in %, temperature in °C. Pompeii worm copepods at in situ pressure have a LD50 of 26.9 ± 0.9°C for 2 h exposures and at atmospheric pressure have a LD50 of 24.9 ± 0.2°C for 2 h exposures. For curve fitting statistics see Table 1. (TIF) [file pone.0333996.s003.tif]
