# Supplementary material for: Limits of life: Thermal tolerance of deep-sea hydrothermal vent copepods and implications for community succession
Source: PLoS One. 2025 Nov 5;20(11):e0333996. doi: 10.1371/journal.pone.0333996 (PMC12588466; doi:10.1371/journal.pone.0333996)
Supplement: S1 Table — (DOCX) [file pone.0333996.s004.docx]

| \| **Pompeii** \| **2h** \| **4h** \| **8h** \| **10h** \|  \| **T&M** \| **2h** \| **4h** \| **8h** \| **10h** \| \| --- \| --- \| --- \| --- \| --- \| --- \| --- \| --- \| --- \| --- \| --- \| \| **4°C** \| X \| X \| X \| X \|  \| **4°C** \| X \| X \| X \| X \| \| **12°C** \|  \|  \| X \| X \|  \| **12°C** \|  \|  \| X \| X \| \| **22°C** \|  \|  \| X \|  \|  \| **22°C** \| X \| X \| X \| X \| \| **25°C** \| X \| X \| X \| X \|  \| **25°C** \| X \| X \| X \| X \| \| **28°C** \| X \| X \| X \| X \|  \| **28°C** \| X \| X \| X \| X \| \| **31°C** \| X \| X \| X \| X \|  \| **31°C** \| X \| X \|  \|  \| \| **34°C** \| X \| X \|  \|  \|  \| **34°C** \| X \|  \|  \|  \| \| **37°C** \| X \| X \|  \|  \|  \| **37°C** \|  \|  \|  \|  \| \| **40°C** \| X \|  \|  \|  \|  \| **40°C** \|  \|  \|  \|  \| \| **43°C** \| X \|  \|  \|  \|  \| **43°C** \|  \|  \|  \|  \| |
| --- | --- | --- | --- | --- | --- | --- | --- | --- | --- | --- | --- | --- | --- | --- | --- | --- | --- | --- | --- | --- | --- | --- | --- | --- | --- | --- | --- | --- | --- | --- | --- | --- | --- | --- | --- | --- | --- | --- | --- | --- | --- | --- | --- | --- | --- | --- | --- | --- | --- | --- | --- | --- | --- | --- | --- | --- | --- | --- | --- | --- | --- | --- | --- | --- | --- | --- | --- | --- | --- | --- | --- | --- | --- | --- | --- | --- | --- | --- | --- | --- | --- | --- | --- | --- | --- | --- | --- | --- | --- | --- | --- | --- | --- | --- | --- | --- | --- | --- | --- | --- | --- | --- | --- | --- | --- | --- | --- | --- | --- | --- | --- | --- | --- | --- | --- | --- | --- | --- | --- | --- | --- |
