# Supplementary material for: Limits of life: Thermal tolerance of deep-sea hydrothermal vent copepods and implications for community succession
Source: PLoS One. 2025 Nov 5;20(11):e0333996. doi: 10.1371/journal.pone.0333996 (PMC12588466; doi:10.1371/journal.pone.0333996)
Supplement: S2 Table — Rhogobius contractus and R. rapunculus could not be separated reliably and are thus labelled together as “Rhogobius sp. (contractus/rapunculus)”. (DOCX) [file pone.0333996.s005.docx]

| **Taxonomic identification** | **PW Habitat** | | **TM Habitat** | |
| --- | --- | --- | --- | --- |
|  | **Total** | **Total (%)** | **Total** | **Total (%)** |
| *Aphotopontius acanthinus* Humes & Lutz, 1994 | 1 | 0.08 | 18 | 1.98 |
| *Aphotopontius arcuatus* Humes, 1987 |  |  | 6 | 0.66 |
| *Aphotopontius limatulus* Humes, 1987 |  |  | 5 | 0.55 |
| *Aphotopontius mammillatus* Humes, 1987 | 4 | 0.33 | 291 | 31.94 |
| *Ceuthoecetes acanthothrix* Humes, 1987 | 15 | 1.24 | 328 | 36.00 |
| *Ceuthoecetes aliger* Humes & Dojiri, 1980 |  |  | 4 | 0.44 |
| *Ceuthoecetes cristatus* Humes, 1987 |  |  | 1 | 0.11 |
| *Ceuthoecetes introversus* Humes, 1987 |  |  | 3 | 0.33 |
| *Exrima dolichopus* Humes, 1987 |  |  | 1 | 0.11 |
| *Exrima singula* Humes, 1987 |  |  | 2 | 0.22 |
| *Nilva torifera* Humes, 1987 | 6 | 0.49 | 56 | 6.15 |
| *Rhogobius pressulus* Humes, 1989 |  |  | 3 | 0.33 |
| *Scotoecetes introrsus* Humes, 1987 |  |  | 15 | 1.65 |
| *Stygiopontius appositus* Humes, 1989 | 63 | 5.19 | 4 | 0.44 |
| *Stygiopontius hispidulus* Humes, 1987 | 1110 | 91.51 | 100 | 10.98 |
| *Stygiopontius paxillifer* Humes, 1989 | 2 | 0.16 |  |  |
| *Stygiopontius sentifer* Humes, 1987 | 7 | 0.58 | 1 | 0.11 |
| *Stygiopontius verruculatus* Humes, 1987 | 1 | 0.08 |  |  |
| *Halectinosoma* sp.1 Vervoort, 1962 |  |  | 1 | 0.11 |
| *Idomene* sp.1 Philippi, 1843 |  |  | 1 | 0.11 |
| *Rhogobius* sp. *(contractus/rapunculus)* Humes, 1987 |  |  | 48 | 5.27 |
| *Stygiopontius* sp.1 Humes, 1987 | 3 | 0.25 |  |  |
| *Tisbe* sp.1 Lilljeborg, 1853 | 1 | 0.08 |  |  |
| Copepodite |  |  | 9 | 0.99 |
| Dirivultid Copepodite |  |  | 11 | 1.21 |
| Dirivultid sp.1 |  |  | 1 | 0.11 |
| Unknown (missing) |  |  | 2 | 0.22 |
